# Influences of polymorphic variants of *DRD2 *and *SLC6A3 *genes, and their combinations on smoking in Polish population

**DOI:** 10.1186/1471-2350-10-92

**Published:** 2009-09-17

**Authors:** Alicja Sieminska, Krzysztof Buczkowski, Ewa Jassem, Marek Niedoszytko, Ewa Tkacz

**Affiliations:** 1 Department of Allergology, Medical University of Gdansk, 7 Debinki, 80-952 Gdansk, Poland; 2 Department of Family Medicine, Nicolaus Copernicus University in Torun, Collegium Medicum in Bydgoszcz, 9 Sklodowska-Curie, 85-094 Bydgoszcz, Poland; 3 Department of Pneumonology, Medical University of Gdansk, 7 Debinki, 80-952 Gdansk, Poland

## Abstract

**Background:**

Polymorphisms in dopaminergic genes may influence cigarette smoking by their potential impact on dopamine reward pathway function. *A1 *allele of *DRD2 *gene is associated with a reduced dopamine D2 receptor density, and it has been hypothesised that *A1 *carriers are more vulnerable to smoking. In turn, the 9-repeat allele of dopamine transporter gene (*SLC6A3*) has been associated with a substantial reduction in dopamine transporter, what might result in the higher level of dopamine in the synaptic cleft, and thereby protective role of this allele from smoking. In the present study we investigated whether polymorphic variants of *DRD2 *and *SLC6A3 *genes and their combinations are associated with the smoking habit in the Polish population.

**Methods:**

Genotyping for *Taq*I*A *polymorphism of *DRD2 *and *SLC6A3 *VNTR polymorphism was performed in 150 ever-smokers and 158 never-smokers. The association between the smoking status and smoking phenotypes (related to the number of cigarettes smoked daily and age of starting regular smoking), and genotype/genotype combinations was expressed by ORs together with 95% CI. Alpha level of 0.05, with Bonferroni correction whenever appropriate, was used for statistical significance.

**Results:**

At the used alpha levels no association between *DRD2 *and *SLC6A*3 genotypes and smoking status was found. However, *A1 *allele carriers reported longer abstinence periods on quitting attempts than non-carriers (p = 0.049). The ORs for heavier smoking were 0.38 (0.17-0.88), p = 0.023, and 0.39 (0.17-0.88), p = 0.021 in carriers compared to non-carriers of *A1 *or *9 allele, respectively, and the OR for this smoking phenotype was 8.68 (2.47-30.46), p = 0.0005 for the *A1*-/*9*- genotype combination, relatively to the *A1*+/*9*+. Carriers of *9 allele of *SLC6A3 *had over twice a lower risk to start smoking before the age of 20 years compared to non-carriers (sex-adjusted OR = 0.44; 95% CI: 0.22-0.89; p = 0.0017), and subjects with *A1-/9- *genotype combination had a higher risk for staring regular smoking before the age of 20 years in comparison to subjects with *A1+/9+ *genotype combination (sex-adjusted OR = 3.79; 95% CI:1.03-13.90; p = 0.003).

**Conclusion:**

Polymorphic variants of *DRD2 *and *SLC6A3 *genes may influence some aspects of the smoking behavior, including age of starting regular smoking, the level of cigarette consumption, and periods of abstinence. Further large sample studies are needed to verify this hypothesis.

## Background

Tobacco smoking is a complex behaviour determined both by genetic and environmental factors. Classical twin studies demonstrated a strong heritability in the range of 40-70%, supporting a substantial genetic contribution to tobacco use [1,2]. Allelic association studies conducted from the beginning of the 1990s have indicated that polymorphisms in several candidate genes may predispose to smoking initiation and nicotine dependence [3]. Recent studies on tobacco smoking have focused on polymorphisms of genes involved in the neurotransmission of dopamine in the central nervous system. The rationale for such an approach ensues from the properties of nicotine as the major addictive substance in tobacco smoke [4]. The pleasurable effects arising from nicotine are linked to stimulation of the dopamine reward pathway after binding nicotine with receptors present on the dopaminergic cell bodies, which in turn leads to an increased release of dopamine in the *nucleus accumbens *of the mesolimbic system [5,6].

A number of allelic association studies focused on polymorphism of gene for *DRD2 *dopamine receptor. Several polymorphisms of this gene were identified, including *Taq*I*A *polymorphisms [7]. Studies have shown that *A1 *allele is associated with a reduced dopamine D2 receptor density [8-10]. It has been hypothesised that persons with such a functional deficit in the dopamine reward pathway experience an enhanced reward when exposed to dopaminergic agents, including nicotine, thereby making them more vulnerable to nicotine addiction [11]. Some authors have demonstrated that cigarette smokers more frequently carry *A1 *allele than never-smokers [11-13]. However, there is some inconsistency in the literature regarding association of *Taq*I polymorphism of *DRD2 *and smoking, with some studies indicating the lack of such an association [14-16] and others reporting the association of *A2 *allele with smoking [17]. More recently, common *Taq*I*A *polymorphism, originally thought to be in the *DRD2 *gene, has been determined to be in a neighbouring gene ANKK1 (*ankyrin repeat and kinase domain controlling 1*) [18]. However, being in linkage disequilibrium with other variants in the *DRD2 *gene, but not with variants in the *ANKK1 *gene, *Taq*I*A *polymorphism is considered to have a strong association with *DRD2 *gene [19].

The dopamine transporter gene (*SLC6A3*) encodes a protein that regulates synaptic levels of dopamine in the brain and has been studied as another candidate gene for tobacco smoking [20-23]. A 40 base polymorphism of a variable number of tandem repeats (VNTR) has been described in the 3' untranslated region of this gene [24]. Initially, it was suggested that people who carry the less frequent nine-repeat allele (*9R) of the dopamine transporter gene are less likely to be smokers [20,23]. The 9-repeat allele of *SLC6A3 *has also been associated with a substantial reduction in dopamine transporter protein, resulting in less clearance and greater bioavailability of dopamine [25]. It was suggested that carriers of the common allele of *SLC6A3*, the 10-repeat allele, may achieve a greater reward from nicotine's effect on dopamine activity. Thus the 9-repeat allele and eventually the higher level of dopamine in the synaptic cleft appeared to play a protective role from smoking. However, it was subsequently demonstrated that the 9-repeat allele enhances transcription of the dopamine transporter protein, which might result in a greater clearance of synaptic dopamine, and thus lower basal availability [26,27]. In turn, some reports suggest that there is no functional difference related to particular polymorphism [28]. There are also some allelic association studies which did not replicate the initial positive results [21,22].

In the light of existing discrepancies and of the lack of surveys on the association between variant alleles of *DRD2 *and *SLC6A3 *genes and smoking in Poland, we investigated whether polymorphic variants of these genes and their combinations are associated with the smoking habit in the Polish population.

## Methods

### The study sample and measures

Three hundred and ten adult subjects (150 ever-smokers and 160 ethnically and gender matched never-smoking controls), Caucasians, were recruited for study among patients and staff of the Academic Clinical Centre in Gdansk and outpatients of the Department of Family Medicine, the Nicolaus Copernicus University of Torun, Collegium Medicum in Bydgoszcz (NCUT-CMB). All subjects were of Polish origin, inhabitants of the Pomeranian region of Northern Poland. Smoking status was elicited before recruitment to the study during face-to-face interviews. Ever-smokers were defined as individuals who have smoked at least 100 cigarettes in their lifetime, and current smokers were defined as individuals who, at the time of the survey, smoked cigarettes either daily or occasionally [29]. Never smoker was someone, who either had never smoked at all or had never been daily smoker and had smoked less than 100 cigarettes (or the equivalent amount of tobacco) in his lifetime [29]. Former smokers were defined as those who had quit smoking at least 1 year before the study. Written informed consent was obtained from all subjects prior to participation in the study, and all study procedures were approved by the institutional research ethics committees at the Medical University of Gdansk and the Nicolaus Copernicus University of Torun.

To verify recent non-smoking status, the measurement of carbon oxide in exhaled air was performed in former smokers with the use of *Micro CO *smokelyser (Bedfont Instruments, Kent, UK). Participants completed a short questionnaire assessing sociodemographic data (age, gender, educational level) and details on the subject's recent and prior tobacco use (ever-/never-smoker status, number of cigarettes smoked, age when regular smoking started, number of years smoked). Current smokers were queried about the longest period of abstaining from cigarettes in the past, if any. All reported periods of maximal abstinence were further calculated into days. Pack-years were calculated using the average number of cigarettes smoked daily and the number of years smoked. Nicotine dependence was scored with the use of the Fagerstrom Test for Nicotine Dependence (FTND) [30].

From all subjects, 8 ml of venous blood samples were collected into heparinised tubes. Samples were frozen and stored at -80°C until required for molecular genotypic analyses. Genomic DNA was extracted from lymphocytes and used as a template for the PCR.

### Genotyping

*Taq*I *A *sites of the *DRD2 *genotypes were determined by polymerase chain reaction-restriction fragment length polymorphism (PCR-RFLP), as previously described [31,32]. The set of primers used was as described by Noble et al. [33].

Allelic variants of *SLC6A3 *gene were characterised by determining a 40-base-pair variable-number tandem repeat in the 3' untranslated region on chromosome 5p16.3 according to Vanderberg et al. [24]. DNA amplification by PCR of the 40 bp repeat was carried out as described by Sano et al. [34]. The set of primers used was as described by Vanderberg et al. [24].

### Statistical analyses

The chi-squared (χ^2^) test was used to assess deviations of genotype distribution from the Hardy-Weinberg equilibrium and for group comparisons of frequencies of allele and genotype/genotype combinations. For analyses, we grouped subjects as carriers and non-carriers, with carriers defined as participants who tested positive for the presence of the allelic variants (*A1/A1 *+ *A1/A2 *and *9/9+9/10+9/11*, versus *A2/A2 *and *10/10+10/11*, respectively) [7,20]. Univariate logistic regression analysis was performed to search for variables potentially associated with the increased risk for ever smoking, i.e. age and education level (gender was not considered in this analysis because of the gender-matching of the study sample), as well as variables potentially increased with heavier smoking (smoking ≥10 cigarettes daily) and earlier starting of regular smoking (before the age of 20 years), i.e. age, sex, and education level. Multivariate logistic regression was performed to examine whether given genotype/genotype combinations were independently associated with an increased risk of particular phenotypes. The association between smoking status or smoking-related phenotypes and genotype/genotype combinations was expressed by ORs with 95% confidence bounds. Student's test t was used to compare means for continuous variables (the U Mann-Whitney test was used where data were not normally distributed). A significance level of 0.05 was set for a type 1 error in all analyses, exluding those, in which Bonferroni correction was needed.

## Results

Out of 310 subjects, two unsuccessfully underwent genotyping, therefore they were excluded from the analyses. Data for the remaining 308 subjects were analysed, including 150 ever-smokers (67 females; mean age 53.1 ± 11.1) and 158 never-smokers (79 females; mean age 45.0 ± 16.2). Distributions of genotypes for *Taq*I*A *polymorphism of *DRD2 *and *SLC6A3 *VNTR polymorphism did not deviate to any appreciable extent from expectations predicted by the Hardy-Weinberg equilibrium as determined by a chi-square test (p = 0.66 and p = 0.36, respectively).

Allele frequencies for the *DRD2 *and the *SLC6A3 *genes and genotypes in relation to smoking status are shown in Table 1.

**Table 1:** *DRD2 *and *SLC6A3 *genotype distribution in ever smokers and never smokers from the North of Poland

**Allele/Genotype**	**Total No. of alleles/subjects**	**%**	**Ever-smokers**	**%**	**Never-smokers**	**%**
*DRD2*:						
*A1*^†^	118	19.2	47	15.7	71	22.5
*A2*	498	80.8	253	84.3	245	77.5
*A2/A2*	204	66.2	106	70.7	98	62.0
*A1/A2*	90	29.2	41	27.3	49	31.0
*A1/A1*	14	4.5	3	2.0	11	7.0
*SLC6A3*						
*9	138	22.4	67	22.3	71	22.5
*10	474	76.9	230	76.7	244	77.2
*11	4	0.6	3	1.0	1	0.3
*10/10*	178	57.8	86	57.3	92	58.2
*9/10*	115	37.3	56	37.3	59	37.3
*9/9*	11	3.6	5	3.3	6	3.8
*11/10*	3	1.0	2	1.3	1	0.6
*11/9*	1	0.3	1	0.7	0	0

^† ^p = 0.032 in comparison of allele frequencies in ever- and never smokers

There were no differences in the distribution of *DRD2 *and *SLC6A3 *genotypes or in the frequencies of *9 alleles of *SLC6A3 *compared to pooled *10 and *11 alleles among ever- and never-smokers. In turn, we found that frequency of *A1 *alleles was lower in ever-smokers than in never-smokers (p = 0.032). However, the OR for being an ever-smoker for *A1 *allele carriers did not reach the assumed level of statistical significance, when compared to *A2 *homozygous subjects and was 0.68 (95% CI: 0.42-1.09); p = 0.108.

Similarly, distribution of genotype combinations did not differ between ever- and never-smokers. Only 41.5% of subjects with a genotype combination *A1*+/*9- *were ever-smokers. A relative risk for this genotype combination for ever-smoking as compared to subjects of all other genotype combinations pooled was 0.69 (95% CI: 0.40-1.21); p = 0.192. In turn, the genotype combination *A1*-/*9*- was the most strongly associated with ever-smoking status: 52.6% of carriers of this variant combination were ever-smokers. However, OR for smoking for subjects of genotype combination *A1*-/*9*- compared to subjects of all other genotype combinations pooled, as well as compared to the genotype combination that had the lowest frequency of smoking (*A1*+/*9*-), did not reach statistical significance and were 1.28 (95% CI: 0.81-2.04), p = 0.288, and 1.56 (95% CI: 0.84-2.80), p = 0.152, respectively.

Comparison of mean values of smoking duration, age when starting regular smoking, the number of cigarettes smoked daily and the number of pack-years between ever-smokers with DRD2*A1 or SLC6A3*9, and those homozygous for DRD2*A2 or SLC6A3*10 demonstrated no differences. There were also no differences when FTND scores for current smokers carrying *A1 *or *9 allele were compared with those for non-carriers. Comparison of mean length of the longest abstinence period on quitting attempts between current smokers with DRD2*A1 or SLC6A3*9 allele and non-carriers for these alleles demonstrated that *A1 *allelic subjects were more likely to achieve longer abstinence than non-carriers of *A1 *allele (314 ± 696 days v. 289 ± 925 days, p = 0.049), while no difference was observed between *9 allele carriers and non-carriers. In the smokers group quantitative measures of smoking did not also relate to *DRD2*x*SLC6A3 *genotype combinations.

Further, multiple analyses with the use of Bonferroni correction were performed to assess the association between the *DRD2 *and *SLC6A3 *genotypes, as well as *DRD2*x*SLC6A3 *genotype combinations and separate phenotypes of ever-smokers related to the number of cigarettes consumed (subjects smoking up to 10 cigarettes/day or occasionally vs heavier smokers), and the age at which regular smoking had started (subjects who had started regular smoking before the age of 20 years versus subjects starting regular smoking being twenty years old or older). We found lower rates of *A1 *and *9 allele carriers in heavier smokers than in light/occasional smokers (68% vs 85%; p = 0.02, and 71% vs 86%; p = 0.02, respectively). Univariate logistic regression analysis demonstrated that the risk of smoking more than 10 cigarettes daily did not relate to age, sex or education level, while *A1/A1+A1/A2 *and *9/9 *+*9/10 *genotypes appeared to decrease it (OR = 0.38 and OR = 0.39, respectively (Table 2). Furthermore, a rate of *9 allele carriers among subjects who had started regular smoking before the age of 20 years was lower than among subjects who had started regular smoking later (51% vs 32% p = 0.022). Univariate logistic regression analysis showed that the risk of earlier transition of initial smoking into regular habit was lower in women and *9 allele carriers (OR = 0.40; 95% CI: 0.21-0.80 and OR = 0.46; 95% CI: 0.23-0.90, respectively), and did not relate to age and education level. Multivariate analysis, with sex incorporated as a covariate, revealed that *9 allele carriers had over twice a lower risk (sex-adjusted OR = 0.44; 95% CI: 0.22-0.89) of starting smoking up to 20 year of life than non-carriers. Stratification by sex revealed that this association reached assumed significance level in males (OR = 0.29; 95% CI: 0.11-0.74), but not in females (OR = 0.80; 95% CI: 0.27-2.35) (Table 2).

**Table 2:** Odds ratios for *DRD2 *and *SLC6A3 *genotypes for separate phenotypes of smoking

**Smoking phenotype**	**ORs (95% CI)**					
	
	***DRD2 *genotypes**		***p*-value**	***SLC6A3 *genotypes**		***p*-value**
	
	***A1*+**	***A1*-**		*9+	*9-	
Ever smoking	0.68 (0.42-1.09)	1.47 (0.91-2.38)	0.108	1.00 (0.65-1.57)	0.99 (0.85-1.15)	0.972

Smoking >10 cig./day	0.38 (0.17-0.88)	2.63 (1.14-6.05)	0.0237*	0.39 (0.17-0.89)	2.59 (1.13-5.93)	0.0211*

Regular smoking before the age of 20 years	0.78^† ^(0.37-1.64)	1.29^† ^(0.61-2.69)	0.022*	0.44^† ^(0.22-0.89)	2.27^† ^(1.09-4.50)	0.00175*
Females				0.80 (0.27-2.35)	1.25 (0.43-3.67)	0.677**
Males				0.29 (0.11-0.74)	3.45 (1.36-8.79)	0.007**

^† ^sex-adjusted ORs

* significance level after Bonferroni correction: p = 0.05/2 = 0.025

** significance level after Bonferroni correction: p = 0.05/3 = 0.017

An estimation of the relationship between genotype combinations and particular smoking-related phenotypes demonstrated that subjects with *A1*-/*9*-, *A1*-/*9*+ and *A1*+/*9*- genotype combinations had nearly 8.7, 4.5, and 4.9 higher risk of being heavier smokers as compared to subjects with *A1*+/*9*+ genotype combination (p = 0.0006, p = 0.014 and p = 0.02, respectively), and subjects with *A1*-/*9*- genotype combination had nearly 4.0 higher risk to step into regular smoking before the age of 20 years as compared to *A1*+/*9*+ genotype status (sex-adjusted OR = 3.79; 95% CI: 1.03-13.90; p = 0.04). Stratification by sex indicated possible association between *A1*-/*9*- genotype and starting regular smoking before the age of 20 years in males (OR = 7.64; 95% CI: 1.29-45.15) but not in females (OR = 1.33; 95% CI: 0.20-8.70) (Table 3).

**Table 3:** Numbers and frequencies of *DRD2/SLC6A3 *combinations, and odds ratios for these genotype combinations for separate smoking phenotypes

**Genotype combinations**	**Light/Heavy smoking** **No. (%)**		**OR (95% CI)** ***p*-value**	**Early/late start of smoking** **No. (%)**		**OR (95% CI)** ***p*-value**
				
	**≤10 cig./day**	**>10 cig./day**		**Before 20 year of life**	**In/after 20 year of life**	
*A1+/9+*	9 (52.9)	8 (47.1)	1^†^	4 (23.5)	13 (76.5)	1^†^
*A1+/9-*	5 (18.5)	22 (81.5)	4.95 (1.22-20.08) 0.017*	13 (48.1)	14 (51.9)	3.02 (0.75-12.13) 0.096*
*A1-/9+*	9 (20)	36 (80)	4.5 (1.32-15.32) 0.013*	16 (35.6)	29 (64.4)	1.79 (0.49-6.59) 0.357*
*A1-/9-*	7 (11.5)	54 (88.5)	8.68 (2.47-30.46) 0.0005*	32 (52.5)	29 (47.5)	3.79 (1.03-13.90)^††^ 0.003*
Females						1.33 (0.20-8.70) 0.865**
Males						7.64 (1.29-45.15) 0.007**

^†^OR = 1 for genotype combination related to the lowest frequency of given smoking-related phenotype

^††^sex-adjusted OR

*significance level after Bonferroni correction: p = 0.05/2 = 0.025

**significance level after Bonferroni correction: p = 0.05/3 = 0.017

## Discussion

Initial studies suggested that *A1 *alleles of dopamine receptor D2 gene were associated with susceptibility to smoking [11,12]. However, several studies failed to replicate this association [14,15], while other studies on Japanese populations indicated the link of *A2 *allele with smoking behavior [17,35].

The results of our study demonstrated no association between *DRD2 *genotypes and cigarette smoking. However, the frequency of *A2 *allele was significantly higher in ever-smokers than in never-smokers. These findings might suggest that *A2 *allele is more important as determinants of smoking status, or that other factors, such as socioeconomic, environmental or cultural ones, might contribute relatively more than *DRD2 *genotypes in the smoking habit among the Polish population.

In our retrospective analysis of quitting history in ever-smokers we found that *A1 *allele carriers achieved longer abstinence periods when they had attempted to quit than non-carriers. However, this association was weak and one could not rule out - false positive, considering the limited sample size of the study and used threshold of *p *= 0.05. Similarly, Spitz et al. [32] in the study conducted in the sample of 283 subjects demonstrated that individuals with *A1 *genotypes reported a slightly longer mean duration of smoking cessation than subjects with the *A2 *genotypes. Other studies, however, yielded inconsistent results regarding a possible link of dopaminergic genes polymorphism with smoking cessation [36-38].

The present study also failed to replicate the association between *SLC6A3 *genotypes and smoking status. Similarly, we found no difference in mean duration of the longest abstinence achieved in the past between allele *9 carriers and non-carriers. Available data on the influence of *SLC6A3 *gene and smoking cessation are also discordant. Erblich et al. found that non-carriers of nine-repeat allele could abstain from cigarettes for a longer period of time than carriers [13]. In contrast, Lerman et al. reported association between the absence of the nine-repeat allele and shorter quit durations [20].

Reasons for non-replication of allelic association studies in general were previously widely discussed [21,39]. It was suggested, for instance, that more attention should be focused on refining smoking behaviour phenotypes and exploring genetic influences on them, rather than only on smoking status *per se *[39]. In fact, only when separating smokers according to their smoking phenotypes related to the number of cigarettes smoked daily and age at starting regular smoking, the possible association between studied polymorphisms and heavier smoking or earlier entering the phase of regular smoking was demonstrated in our investigation. It is likely that other genes than *DRD2 *and *SLC6A3 *have a greater impact on ever smoking status (i.e., smoking initiation), and simultaneously many other non-genetic factors, including socio-demographical, pharmacological, psychological, behavioural or cultural ones, independently influence vulnerability to smoking, while these two dopaminergic genes might influence smoking behaviour phenotypes. However, it is possible, that the association between studied polymorphisms and these smoking-related phenotypes was mediated by an unknown confounder, for instance, linkage disequilibrium between a variant under study and true variant related to given smoking-related phenotype. On the other hand, it is likely that ethnical homogeneity of studied population allowed to avoid confounding by ethnicity.

Our survey on association between genotypes and the level of cigarette consumption demonstrated that the risk for smoking a greater number of cigarettes daily was over 2.5 times lower in carriers than in non-carriers of *A1 *or *9 allele. Moreover, frequency of heavier smoking was the lowest among subjects with *A1+/9+ *genotype combinations. In turn, possessing even one common allele *A2 *or *10 in *DRD2xSLC6A3 *combinations was associated with a higher risk of heavier smoking, with *A1-/9- *genotypes combination associated with the highest risk of heavier smoking, relatively to the *A1+/9+ *genotype combination. Previously, Spitz et al. [32] reported that *A1 *allele carriers were less likely to smoke more cigarettes per day than non-carriers. In turn, Yoshida et al. reported the lack of apparent association between *DRD2 *genotype and the number of cigarettes smoked daily [17]. Our study indicated that also genotype combination *DRD2/SLC6A3 *might influence the level of cigarette consumption. Namely, smokers with a relatively regular synaptic cleft dopamine homeostasis might experience a greater reward when stimulated by nicotine and, in consequence, they might be apt to repeat this stimulation more frequently by smoking cigarettes. Assuming the hypothesis that *A1 *carriers have a lower density of DRD2 receptors [8-10] and 9-repeat allele carriers display decreased expression of dopamine transporter [25], individuals with *A1*+/*9*+ genotype combination might have a chronically high level of extra-cellular dopamine protecting them from heavier smoking. In fact, our findings that *A1*+/9+ genotype combination had the lowest association with heavier smoking might be explained by the higher dopamine availability resulting from lower dopamine receptor density accompanied by decreased activity of dopamine transporter, not sufficient to uptake the whole amount of dopamine from the synaptic cleft. Such subjects might have a lower need to repeat dopaminergic stimuli, because of protracted extracellular dopamine signalling.

Separating smokers by the age when they step onto the stage of regular smoking revealed that carriers of *9 allele of *SLC6A3 *were less likely to indurate the smoking habit before the age of 20 years than non-carriers, and that this association was modified by gender. In addition, with combined *DRD2 *and *SLC6A3 *genotypes, subjects with *A1-/9- *genotype combination had a higher risk for staring regular smoking before the age of 20 years in comparison to subjects with *A1+/9+ *genotype combination, with OR reaching in males 7.6. Data from the initial study of Lerman et al. [20] on the association between *SLC6A3 *VNTR polymorphism and smoking indicated that 9-repeat allele carrier genotypes were significantly less likely to have started smoking before 16 years of age. Our data are consistent with the observation that polymorphism in the *SLC6A3 *may play an important role in smoking onset and progression to regular smoking, as well as indicate that gender, independently influencing the age of induration of smoking habit, might be a strong effect modifier.

As a limitations of the study the relatively small study sample should be pointed out. It has resulted in quite wide confidence intervals for subgroups referring to particular smoking phenotypes indicating uncertainty in the result. Despite in several studied associations p-values were smaller than used thresholds we are cautious in interpreting arbitrary these findings as significant, keeping in mind the possibility of false positivity due to chance caused by random variation [40]. Thus, we present our results as hypothesis-generating rather than confirmatory and consider that they need validation in studies conducted on larger sample of smokers. However, although a statistical power of the present study seems to be not adequate, our study indicate an importance of extending the allelic association studies by the investigation of the potential link of candidate genes polymorphisms with better defined smoking-related phenotypes rather than only with smoking status *per se*.

## Conclusion

Polymorphic variants of *DRD2 *and *SLC6A3 *genes might influence some aspects of the smoking behavior, including age of starting regular smoking, the level of cigarette consumption, and periods of abstinence.

It might help in identifying special subgroups of smokers, which, in turn, could have important clinical implications for more successful interventions. It is likely, however, that due to the great complexity of smoking behaviour, which results from numerous genetic, environmental and psychological influences, the knowledge of genetic risk alone could be insufficient for the development of more effectively targeted cessation therapy.

## Competing interests

The authors declare that they have no competing interests.

## Authors' contributions

AS conceived the study, participated in its design and coordination, and drafted the manuscript. KB participated in the data collection phase, helped to interpret findings and contributed to the text. EJ participated in the design, coordination and supervision of the study and helped to draft the manuscript. MN performed the statistical analysis. ET participated in the data collection phase. All authors reviewed drafts of the manuscript and approved the final version before submitting it for publication.

## Pre-publication history

The pre-publication history for this paper can be accessed here:


